# Effect of Thyroid Dysfunctions on Blood Cell Count and Red Blood Cell Indice

**Published:** 2013-04-22

**Authors:** A Dorgalaleh, M Mahmoodi, B Varmaghani, F Kiani node, O Saeeidi Kia, Sh Alizadeh, Sh Tabibian, T Bamedi, M Momeni, S Abbasian, Z Kashani Khatib

**Affiliations:** 1 Hematology Department Allied Medical School, Tehran University of Medical Sciences, Tehran, Iran.; 2 Pars pathobiology laboratory, Minoodasht, Golestan, Iran.; 3 Medical Laboratory Sciences Department Allied Medical School, Zahedan University of Medical Sciences, Zahedan, Iran.; 4 Parasitology Department Allied Medical School, Zahedan University of Medical Sciences, Zahedan, Iran.; 5 Imam Ali hospital, Sistan and Baluchistan, Chahbahar, Iran.

**Keywords:** Hypothyroidism, Hyperthyroidism, TSH

## Abstract

**Background:**

Thyroid hormones have a crucial role in metabolism and proliferation of blood cells. Thyroid dysfunction induces different effects on blood cells such as anemia, erythrocytosis leukopenia, thrombocytopenia, and in rare cases causes’ pancytopenia. It also alter RBC indices include MCV, MCH, MCHC and RDW. Thus this study attempted to evaluate effect of hypo & hyperthyroidism on blood cell count and RBC indices.

**Materials and Methods:**

This study performed on 102 patients with hypothyroid (14.1 years), 84 with hyperthyroid (15.6 years) and 118 healthy individuals (15.2 years) as control group. Initially patients TSH level of patients was determined by ELISA method, and then according to TSH ranges (0.3-5.5µIU/mL) patients

were divided into two Hyperthyroidism (TSH<0.3µIU/mL) and hypothyroidism (TSH>5.5µIU/mL) groups. Then, complete blood count was measured by cell counter. Finally, obtained results were analyzed by SPSS software.

**Results:**

Analyzes of obtained data revealed statistically significant difference between two groups of patients in RBC count, MCH, MCHC, RDW, HB and HCT(P-value<0.05), but the difference was not significant for WBC and PLT counts and MCV (P-value>0.05).

**Conclusion:**

In case of patients with unknown hematological dysfunctions, must be evaluated for thyroid hormones.

## Introduction

The thyroid gland, which is the largest and important endocrine gland in human body, is located on the anterior side of the neck, right below the larynx. It has two lobes and is composed of many thin follicular cells with a type of epithelial tissue origin. These follicles store thyroid hormones in the form of Thyroglobulin molecules until body requires them. Thyroid gland synthesizes and secrets two major hormones, known as 3, 5, 3’-triiodothyronine (T3) and thyroxin, which can sometimes be referred to as 3, 5, 3’, 5’-tetraiodothyronine (T4). These hormones are necessary for mediate metabolism rate (how quickly the body uses and stores energy). These molecules have also critical roles in early brain development, somatic growth, bone maturation, protein synthesis and regulating production of red blood cells. All these functions are regulated by attachment of the active form of thyroid hormone T3 to specific members of the nuclear receptors family (TRa and TR_B_). Hormonal output from the thyroid is mediated by thyroid stimulating hormone (also known as TSH or thyrotropin) secreted by anterior pituitary. The secretion of thyrotropin itself is mediated by thyrotropin-releasing hormone (TRH) secreted by the hypothalamus. Other effects of thyroid hormones include involvement in hemoglobin production in adult and maturation of Hb in fetus (2-4). The most common disorders of the thyroid gland include hyperthyroidism, hypothyroidism and thyroid nodules, which are generally benign thyroid neoplasm but may change to thyroid cancer. Thyroid disorders are frequently accompanied by red blood cell abnormalities. Thyroid hormones often have important effect on erythropoiesis. They enhance erythropoiesis through hyper proliferation of immature erythroid progenitors and increase secretion of erythropoietin (EPO) by inducing erythropoietin gene expression. Thyroid hormones also augment repletion of hypoxia inducible factor1 (HIF-1) and then motivate growth of erythroid colonies (BFU-E, CFU-E).These hormones also intensify erythrocyte 2, 3 DPG compactness, which enhances the delivery of oxygen to tissues. Hyperthyroidism causes mild decreases in total white blood cell count, neutropenia, thrombocytopenia and increases, normal or mild decreases in total white blood cell count. Generally it seems that hypothyroidism causes hypoplasia in all myeloid cell lineages and hyperthyroidism result in hyperplasia. With regard to lymphocytes, T3 is as a precursor substance for normal B cell formation in bone marrow through its mediation of pro-B cell proliferation. Therefore, thyroid disorders can induce different effects on various blood cell lineages (7-10). Hypothyroidism can cause various forms of anemia (normochromic-normocytic, hypochromic-microcytic or macrocytic) through reducing the oxygen metabolism. Microcytic anemia generally attribute to malabsorption of Iron and loss of Iron by menorrhagia, whereas, macrocytic anemia causes or induces malabsorption of vitamin B12 , folate, pernicious anemia and insufficient nutrition (10). 

On the other hand, anemia frequently is not seen in patients with hyperthyroidism, while there were erythrocytosis in this situation, but when anemia present, may be morphologically similar to that observed in hypothyroidism. Patients with hypothyroidism have a decreased erythrocyte mass due to reduction of plasma volume and may undetectable by routine measurement such as hemoglobin concentration, whereas an increased erythrocyte mass is observed in most hyperthyroid patients (11-12) . Alteration in other hematological parameters such as hemoglobin (HG), hematocrit (HCT), mean corpuscular volume (MCV) ,mean corpuscular hemoglobin (MCH), white blood cell (WBC) count and platelet count is associated with thyroid dysfunction is observed as well (9), but all changes return to normal if an euthyroid (normal) state is obtained. Pancytopenia is a rare side effect of that its cause is not well understood. Immunological mechanisms have been offered for decline of the life-span of erythrocytes and platelets (13).Because of high prevalence of thyroid dysfunctions in Iranian population, we attempted in the present study to evaluate the effect of thyroid dysfunctions particularly cells and red blood cells indices.

## Materials and Methods

This cross sectional study was conducted on 102 patients with hypothyroidism and 84 patients with hyperthyroidism from June to August 2012(in order to matching two groups in term of age and sex, we had to select a larger group for hypothyroidism patients). All patients who referred to laboratory in this interval, after application of inclusion and exclusion criteria, were enrolled in study. Ethical approve and patients consent statement were taken from all patients. We also chose a group of 118 euthyroid individuals as control.Control group was selected among age and sex matched euthyroid individuals without any thyroid and hematological disorders.

All patients with established thyroid dysfunction were included in the study and then patients with special diseases that could affect red blood cell indices and also with inappropriate samples were excluded from the study.

Reference range for thyroid stimulating factor was 0.03 –5.5µIU/mL, and according to this range we divided patients in to two age and sex matched groups of hypothyroid (TSH> 5.5µIU/mL) and hyperthyroid (TSH <0.3µIU/mL).

Initially two separate blood samples were taken from each patient, 2 ml uncoagulated sample was harvested for assay and Ethylenediaminetetraacetic acid (EDTA) anti coagulated whole blood sample for complete blood cell count.

Serum samples were used to determine level of TSH, T3 and T4 (ELISA) and then complete blood count were measured with EDTA anti coagulated samples by Sysmex (kx 21 Japan).

**Statistical Analysis**

Statistical analysis was performed by SPSS software. Results were reported as mean ± standard deviation (SD) for quantitative variables and percentages for categorical variables.

Statistical Independent T test was used to evaluate the significance of differences between two groups. P-value < .05 was considered as a significant change.

## Results

In the 102 patients with hypothyroidism , mean age was 14.1 years (min 3, max 34) and in 84 hyperthyroidism patients mean age was 15.6 (min 4, max 34) and in control group the mean age was 15.2 years (min 2, max 30) (Table I). Comparison of RBC in two groups of hypothyroidism and hyperthyroidism revealed that most of red blood cell indices including MCH, MCHC, HB, HCT and RDW have significant statistical difference (P-value= 0.0001) but no difference was observed for MCV (P-value >0.05) (Table II). Comparison between control group and two groups of hypothyroidism and hyperthyroidism revealed statistically significant difference in RBC count, HCT, Hb, MCH, MCHC and RDW (P-value<0.05) parameters but no significant difference observed for MCV (P-value >0.05).

**Figure 1:**
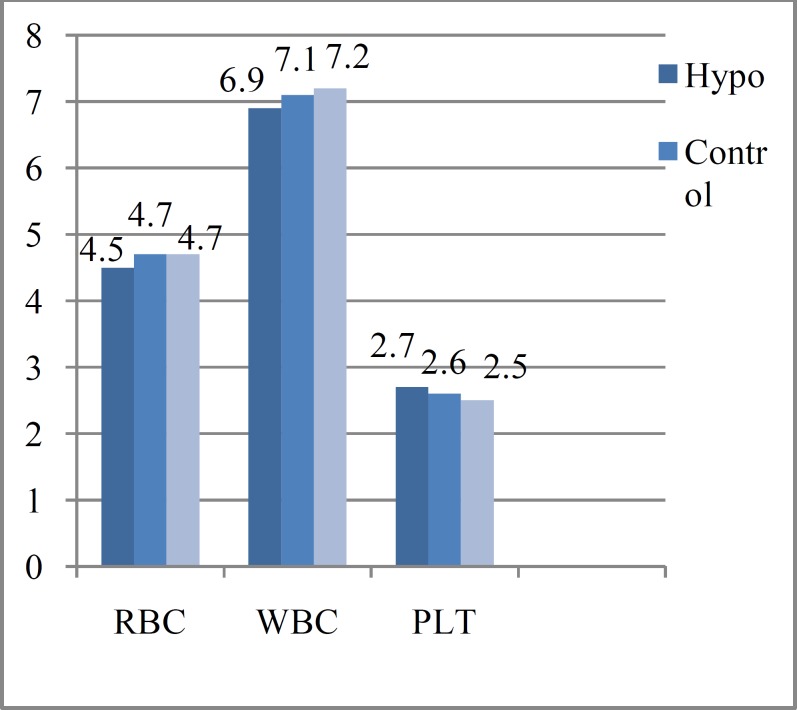
Blood cell counts in hyperthyroidism and hypothyroidism patients. RBC count×10^9^, WBC count×10^3^, PLT count×10^6^

Blood cell count in these two groups of hypothyroidism and hyperthyroidism revealed that just RBC count has statistically significant difference (0.0001), but WBC and PLT counts did not show any statistical significant difference between these two groups (P-value>0.05) (Figure 1). Platelet and WBC counts in both patient groups compared with the control group did not show significant differences. 

**Table I:** Descriptive analysis of patients with hypothyroidism and hyperthyroidism

	**Number**	**Age(mean)**	**Max** **(year)**	**Min** **(year)**	**Male(%)**	**Female(%)**	**TSH (µIU/mL)** **(mean)**	**T3(ng/ml)** **(mean)**	**T4(µg/dL)** **(mean)**
**Hypothyroidism**	102	14.1	34	3	38	62	4.97	1.00	1.16
**Hyperthyroidism**	84	15.6	34	4	42	58	0.18	1.62	11.97
**Control**	118	15.2	30	2	39	61	2.6	0.7	7.2

**Table II:** Comparison between blood cells count and RBC indices in patients with hypothyroidism and hyperthyroidism

Index		Number of Patients	Mean	Std. Deviation	P-Value
**MCV(fl)**	**Hypothyroidism**	102	84	9.87	P1=0.01 P2=0.006
	**Control**	118	85	7.9	
	**Hyperthyroidism**	84	81.7	8.27	
**MCH(pg)**	**Hypothyroidism**	102	27.4	3.95	P1=0.004 P2=0.003
	**Control**	118	29.3	2.9	
	**Hyperthyroidism**	84	27	3.60	
**MCHC(g/d)**	**Hypothyroidism**	102	32.5	1.79	P1<0.001 P2=0.002
	**Control**	118	33.6	1.4	
	**Hyperthyroidism**	84	32.8	1.64	
**RBC ( mil/µL)**	**Hypothyroidism**	102	4.5	0.55	P1=0.06 P>0.05
	**Control**	118	4.7	0.46	
	**Hyperthyroidism**	84	4.7	0.62	
**Hb (g/dL)**	**Hypothyroidism**	102	12.2	1.46	P1<0.0001 P2=0.006
	**Control**	118	13.6	1.17	
	**Hyperthyroidism**	84	12.5	1.40	
**HCT ( % )**	**Hypothyroidism**	102	37.5	3.57	P1=0.004 P2=0.003
	**Control**	118	40.6	2.9	
	**Hyperthyroidism**	84	38.2	3.60	
**RDW (%)**	**Hypothyroidism**	102	13.7	1.73	P1<0.0001 P2<0.0001
	**Control**	118	12.9	1.2	
	**Hyperthyroidism**	84	14.7	11.74	

## Discussion

Thyroid gland as the largest and the most important endocrine gland of human body with the secretion of two hormones, T3 and T4, has a major role in metabolism of cells and organs. Thyroid gland also has a crucial effect on erythropoiesis by induction of erythropoietin secretion and also proliferation of erythroid progenitors (1, 8, and 11).

The most common thyroid dysfunctions, hypothyroidism and hyperthyroidism affect blood cells and cause anemia with different severity. These thyroid disorders also cause thrombocytopenia, leukopenia and even in rare cases cause pancytopenia (in hypothyroidism). Other blood indices including MCV, MCH, MCHC, Hb also could change during thyroid dysfunction (9).

Thus, this study aimed to evaluate effects of thyroid dysfunctions (hypothyroidism and hyperthyroidism) on blood cells count and red blood cells indices.

According to obtained data, red blood cell count was statistically different between patients with hypothyroidism and hyperthyroidism (P-value <0.05), but WBC and PIT count did not show statistically significant different in these two groups of patients (P-value>0.05).

Also most red blood cell indices and parameters including MCH, MCHC, RDW, Hb and HCT had statistically significant difference between two groups of patients (P-value <0.05). RDW in these two groups did not show statistically significant difference (P-value>0.05).

Comparison of two groups of patients (hypothyroidism and hyperthyroidism) with control group revealed statistically significant difference about Hb, HCT, MCV, MCH, MCHC and RDW but no about RBC, WBC and PLT count.

In a study by Geetha J and Srikrishna R in 2012, red blood cell indices were compared in patients with hypothyroidism and hyperthyroidism and revealed that RDW and MCV in these two groups of patients in comparison to euthyroid individuals have statistically significant difference but other RBC parameters like HB and HCT did not show any significant difference in comparison with euthyroid status but in our study, these parameters were statistically different between patients with hypothyroidism and hyperthyroidism and control group except for MCV (14).

Kawa MP and et al in 2010 reported that RBC, HB and HCT in patients with hyperthyroidism were significantly higher than control groups while RBC and HB were decreased in hypothyroidism, while HCT was increased. They also showed that MCH and MCHC were lower in both groups in comparison with control group and MCV was increased in two groups of hypothyroidism and hyperthyroidism (9).

Carmen S.P. Lima and et al in 2006 described four patients with Graves’ disease who had severe pancytopenia. Finally they concluded that thyroid evaluation for all patients with pancytopenia should be performed even though no related symptoms are found (13).

According to obtained data we suggested that all patients with hypothyroidism and hyperthyroidism should be periodically evaluated for probably hematological changes.

## Conclusion

Thyroid dysfunctions have a direct effect on most red blood cells indices and these changes should be considered by medical care provider.
